# Trilingual and Multicultural Experiences Mitigating Students’ Linguistic Stereotypes: Investigating the Perceptions of Undergraduates of Chinese Heritage Regarding Native/Non-Native English Teachers

**DOI:** 10.3390/bs13070588

**Published:** 2023-07-14

**Authors:** Minmin Yang, Gretchen McAllister, Bin Huang

**Affiliations:** 1 College of Foreign Languages, Huaqiao University, Quanzhou 362021, China; 2 College of Education, Northern Arizona University, Flagstaff, AZ 86011, USA; gretchen.mcallister@nau.edu

**Keywords:** linguistic stereotype, trilingual, multicultural experiences, Chinese heritage students, native English teachers, non-native English teachers, homophily, World Englishes (WE)

## Abstract

Student stereotyping of non-native English-speaking teachers is a common focus of research and there is a paucity of studies targeting trilingual students of multicultural backgrounds. The present study aims to investigate the dimensions of trilingual Chinese heritage undergraduates’ perceptions of English teachers from Kachru’s stratification of native-English-speaking (Inner-circle), ESL (Outer-circle) and EFL (Expanding-circle) regions. A mixed study design was used to collect data including online questionnaires and an offline semi-structured interview. Quantitative findings indicate the subjects’ preference for native speaking teachers, together with a hierarchical ranking in teacher assessments according to race/ethnicity. Qualitative findings demonstrate that students are also less biased on racial grounds, considering all teachers are “qualified and good enough”, hence the “Inner > Outer > Expanding = Qualified > Unqualified” result. Multilingual and multicultural factors have been used to account for the mitigated linguistic stereotypes from sociocultural and political perspectives. Findings of this study challenge but nevertheless also confirm to some extent the traditional native/non-native dichotomy that manifests raciolinguistic traits and support Kachru’s stratification with statistical evidence. Educational implications are discussed to benefit future practice to further eliminate student prejudice and to better prepare native Chinese teachers of the English language.

## 1. Introduction

With the continued advance of globalization, despite headwinds such as the recent pandemic, tertiary education has become more diverse. This is not only the case in developed Anglophone countries like the US, UK, Canada or Australia, which have long been major destinations for “study abroad” for students worldwide, but also in some developing countries such as China. Some challenges have emerged inherent in international study in multicultural settings which have become new and pressing topics for research. This includes perceptions of teacher qualifications in regard to the teaching of English as a foreign language (EFL).

There is near consensus that native-English-speaking students (NS) in Northern America harbor prejudice against non-native English-speaking teachers (NNS), rating them less favorably and less effective than their NS counterparts, no matter how effectively these teachers are prepared [1,2,3,4,5,6]. Some scholars name this phenomenon “the native speaker fallacy” [7], or “native speakerism” [8]. Indeed, a recent study suggests that this sociolinguistic stereotype can even be detected in Canadian children of only 5 years of age [9]. Researchers have been including the native fallacy and sociolinguistic stereotyping under the discipline of “raciolinguistics”, claiming that “white supremacy” is behind these phenomena [10,11]. Furthermore, the prejudice of students against their teachers may have negative effects on their academic performance and knowledge acquisition [12,13,14,15,16], while a positive teacher–student relationship promotes mental and physical health of students and is a key determinant of achieving positive communication outcomes [17].

Teacher–student relationships, especially in a multicultural context, have been under-researched [18]. As China grows in global stature, an increasing number of Chinese-heritage students have “returned” to mainland China to study and seek their cultural roots. This group of students is typically trilingual—they speak the first language of where they come from in addition to Chinese (as they are required to have a certain level of Chinese mastery before university admission on the mainland) and also English as most of them join English-medium instruction (EMI) courses; in addition, English is a compulsory course in almost all Chinese universities. While voluminous research has been undertaken regarding student acquisition of the Chinese language, which has even become a whole discipline in China, little attention has been paid to student attitude and experience in their English study, let alone their teacher–student relationship with the English teachers. This study, therefore, aims to fill this research gap, to investigate student attitude towards and assessment of their English teachers and to discover whether there is a positive teacher–student relationship in this field; that is to say, whether the students are less biased against the teachers on several communicative and professional variables. The set of students participating in the study also provides us with an interesting lens as insiders/outsiders having experienced learning multiple languages, including English, within a non-Chinese context prior to their arrival to China.

### 1.1. Literature Review

#### 1.1.1. Teacher–Student Homophily

The teacher–student relationship is considered a dyadic social process that involves ongoing interactions of the two parties in classroom settings [19]; interpersonal measures have been introduced into the field, such as the principle of homophily [20,21]. Homophily refers to the degree of similarity between the two parties of communication, and the opposite is heterophily. It is widely believed that a higher level of homophily usually promises a more successful outcome of communication, namely the interpersonal homophily principle [22]. *Science* has reported on the significant effect of homophily on a group’s acquisition of novice health habits [23]. In the case of higher education, Northern American students prefer NS teachers who are from the same ethnic, social or cultural background; these students deemed the NNS teachers “Other” [4], showing a high level of student–teacher heterophily consequently leading to distorted information transfer or compromised effects in academic acquisition [6,13,16,24]. It is difficult to assess the extent of homophily between two groups in reality, and therefore it is common practice for researchers to assess the perceived homophily with questionnaires.

Homophily scales have been utilized to investigate the teacher–student relationship; the correlation between homophily and the student–teacher assessments are often examined. Scholars have found that the teacher–student background and attitude homophily and interpersonal attraction positively relate to instructor’ immediacy [25], an attribute that has been posited to have a linear relationship with increased student learning [26]. A correlation is detected between gender homophily and the period of PhD dissertation completion, student class satisfaction rating of lectures and rate of class participation [27,28]. In a hybrid higher education context, racial/ethnic homophily is a key topic, sharing similar research attention with gender, background, and attitude homophily; it is closely related to and examined alongside the trustworthiness of the instructors, attraction of tasks, and value orientations, exerting a considerable impact on the effects of teaching and studying (e.g., [29,30,31,32]). These studies have proven the reliability and practical significance of applying homophily scales within student–teacher assessment.

A second generation of homophily scales has been proposed and tested with reasonable validity and reliability. This model consists of two dimensions of homophily, attitude and background, and has been proven to work better in line with the homophily principle. Further empirical studies are now anticipated using this second generation of such scales [33].

#### 1.1.2. The Raciolinguistic NNS Stereotype and Reverse Linguistic Stereotype

When undergraduates are asked to rate their teachers, their assessments are every so often influenced by the teachers’ demographic attributes, such as race, language background, or gender [6,9]. Influenced by what Holliday [8] termed the native speaker fallacy, they showed a linguistic stereotype, claiming that the NNS speakers’ English usage is “broken” [5] and that the NS speakers are linguistically and culturally superior to their NNS counterparts [13]. Previous studies have repeatedly tested and revealed the NS students’ prejudice against their NNS teachers (as mentioned in the Introduction). Accented speech was usually equated to poor teaching abilities of an NNS teacher [6,12,34]. Linguistic competence and teaching competence are usually surveyed for the detection of NNS bias.

On hearing samples of merely accented speech, negative attributions are associated with the NNS speaker who is then regarded as having low prestige and rated inferior in many ways, with this being termed a linguistic stereotype. Another pattern has been detected by Kang and Rubin that is termed Reverse linguistics stereotype (RLS) [16], meaning the idea or perception of an NNS identity, or even the face of an NNS, is a predictor strong enough to trigger distorted evaluation of that person. The pattern was more often discovered using the design of matched guise. In this design, the guises (a picture or knowledge of an Asian instructor and those of a Caucasian instructor) were used to color participant perceptions of certain speech types. Through this design, it was observed that while they were actually listening to recordings of an NS disguised as Chinese/Asian, US students simply rated the Chinese face more negatively [6] and the same utterances associated with an Asian face “less intelligible” [35]. Given the influence of race and language in this process, this type of prejudice can actually fall on a much broader theory of raciolinguistics, which claims that linguistic stereotype is one product of the ongoing process of white supremacy [10].

Meanwhile, NNS prejudice and (reverse) linguistic stereotyping has also been detected in students and the education discipline and industry outside the Anglosphere. In China’s mainland, the matched guise method revealed the RSL pattern with the Cantonese learner of English: after listening to the same recordings by an NS speaker, those who were told that they were listening to an NS speaker outperformed those who were told that they were listening to a Cantonese speaker in almost all listening comprehension tasks [15]. Also, with Cantonese learners, a similar pattern was predicted, in which their idealized perception of native-English-speaking teachers resulted in more negative perceptions of the same features in non-native speech [36]. Taking Chinese mandarin-speaking EFL learners as subjects, Yang et al. (2017) [29] copied the matched guise design and coupled it with homophily and multi-dimensional Speech Evaluation Instruments such as questionnaires and interviews, and also pre-, post-, and delayed tests. They explored the homophily effects on acquisition. Results are that learners under-evaluated NNS teachers’ accents merely out of prejudice. Moreover, racial homophily has a negative correlation with L2 student evaluation and leads to statistically significant differences in comprehension test results, which can be a predictor of comprehension scores. In Taiwan, interviews and questionnaires helped to discover that English major graduates are willing to learn different varieties of English concerning accents and word use; however, they still prefer English conforming to NS norms [37].

In South Korea, case studies and content analysis show that the English teaching workforce remains subject to the NS fallacy. Seo (2023) implemented a case study of the racialized experiences of a female Ugandan teacher of English who has an MA degree in TESOL and is an experienced English teacher, identifying herself as an almost native speaker. However, she reported being rejected by the formal accreditation process in Korea and having her qualification as an English teacher scrutinized and challenged [38]. By examining public texts in Korea, Anh (2019) found out that in mainly English immersion camp advertisements and teacher employment materials, nativeness was the very first token for language proficiency, leaving actual teaching qualification only secondary. In addition, raciolinguistics may have played a part here as she was a Black woman NNS [39].

Although some scholars have recently detected that multiculturalism has a mitigating effect on NNS stereotypes (e.g., Refs. [1,4,40]), similar preference for NS teachers still exists in the highly internationalized harbor of Hong Kong: Tsang (2020) explored learner perception of both L1 and L2 accents and found that nativeness of an instructor was more strongly correlated with the suitability for the job than comprehensibility [41]. In another multicultural setting, Boonsuk et al. (2021) commented that native speakerism and EFL-oriented pedagogies still dominate in Thailand, despite the increasing diversity of languages and cultures in the society [42].

As the extant literature makes manifest, NNS prejudice creates at least two substantial negative consequences for students: first, an unfair psychosocial bias detrimental to the mental health and the whole-person education of these students, and second, impediment of both teacher–student relationships and students’ academic performance.

#### 1.1.3. The Challenged NS/NNS Dichotomy and Kachru’s Three-Circle Theory

Although the NS/NNS dichotomy has been well established and closely examined, some studies have challenged its dominance in related research designs. A conclusion is easily drawn from a closer examination of the matched guise design that the underlying bias is based upon not only the NNS stereotype, but also a racial/ethnic ideology, as when the raters prioritize the so-called NS speaker, it is usually a Caucasian face—a “White” identity—that triggers a higher evaluation. For example, in the Alsace region of France, African-American English was associated with positive human characteristics, but also a lack of education [43]. As Ghanem and Kang (2021) pointed out in their discussion of their RLS findings, it is suggested that there is “prejudice against teachers with presumed (East) Asian background, and not necessarily against NNSs as a whole” [12]. This is consistent with a recent empirical study by McDonough et al. (2022), in which it was discovered that Chinese and South Asian students were rated by their Canadian university peers as more accented and less comprehensible than their European counterparts [44].

As previously mentioned, when plotting an NS/NNS-dichotomy-based language attitude or interpersonal relationship investigation, especially in the matched guise scheme, it is more often the racial/ethnic identity that is featured, covered, or guised by its linguistic surface. NS preference itself might be a guise of racial discrimination. As pointed out by Lee and Canagarajah (2019), the binary NS/NNS dichotomy only reifies monolingual ideologies, in spite of all the efforts to empower NNSs [45]. Cheng et al. (2021) argue that the term NS is not valid for the construction of rigorous theories, and is harmful to marginalized populations by reproducing normative assumptions about behavior, experience, and identity [46]. It often omits important details and diversity that researchers need to consider [47]. Also, such a distinction may wrongly presuppose that an NS is a successful speaker while an NNS is less successful in language acquisition, disregarding their needs and abilities [48], while in reality not all native speakers are good English speakers [49]. With the concept of an NS/NNS dichotomy under examination with regard to ambiguity of research design and theoretical rigorousness, a more diverse pattern must be introduced to relevant empirical studies, this being Kachru’s theory of World Englishes (WE) [50].

Kachru (1992) [50] innovatively proposed that “the current sociolinguistic profile of English may be viewed in terms of three concentric circles”: the Inner circle, the Outer circle, and the Expanding circle, which refer, respectively, to the NS countries that are the traditional cultural and linguistic bases of English, ESL regions that have experienced a certain periods of colonization, especially by the Inner circle, and regions where the use of English is essentially considered EFL. Through this triple stratification of the Three Circles, WE obviously provide one more dimension to the original binary distinction and thereby a more realistic significance deriving from the actual uses of English.

This theory has ignited much debate, criticism and challenges, for example, for its implied “correctness” of the English used by the Inner circle [51], the ambiguity of its members [52], or a possible “Fourth Circle” fueled by technology that it could not have expected [53]. Nevertheless, many linguists still consider it one of the most influential models for understanding the use of English worldwide [52].

The Outer-circle SOCs are appropriate subjects to fill the gap of WE studies. Theoretically, research on these students will contribute to the WE literature because they usually have real encounters with teachers of all three Circles, and such research will be trusted to produce real instead of imaginative assessment to teachers; meanwhile, many of EFL students of the mainland only have had local (Expanding) EFL teachers throughout their studies, making them less qualified to comment on the Outer- and Inner-circle teachers. Also, practically, although trilingual speakers might not be difficult to find, it is still always easier for researchers to collect data from a group of students who study and live together. Furthermore, raciolinguistics suggest that race and language should be observed together on a much more “macro context” level, involving political and economic systems, among others [10,11]. Under the influence of Taoist philosophy of “attracting the faraway”, the overseas Chinese, including the SOC students, have long been an important object of naturalization and friendliness of the PRC government. To this end, ever since its founding, the PRC government has set up a unique national department to work for and with them. Much favorable mutual work has been conducted throughout history. As China becomes stronger in economy, more Chinese heritage people choose to come back to China and they enjoy a great number of favorable policies in living and work. It is therefore worthwhile to test whether the policies have been implemented successfully on campus settings.

### 1.2. The Present Study

A number of empirical studies have been implemented to inspect the influences and extents of WE in education. For example, it is revealed that features of Indian and Chinese English, representing the Outer and Expanding circle, respectively, affect the student interpreters’ performance of consecutive interpreting [54], and that Japanese students in the Outer circle in Singapore still have a preference for “native” teachers [55]—it is therefore obvious that there still exists “bias” against NNS despite this triple stratification of WE. Lu et al. (2022) [56] were among the few researchers to adopt the Three-Circle construction as the scaffold for English textbook examination; however, only a limited number of studies have used WE as the scaffold for a student–teacher relationship survey in which different varieties of English are involved.

This present study aims to observe the assessment made by trilingual Chinese heritage students from the Outer-circle regions of their English teachers of the Three Circles, with a triangulation design of the use of homophily scales, teacher assessment scales, and a third-party interview. Given the previous discussion regarding the potential mitigating effect of multiculturalism on NNS stereotypes (Refs. [1,4,40], etc.), this particular group of participants provide a unique opportunity to examine this assumption. The trilingual Chinese heritage students have been exposed and interacting in their home communities with various races, Asian teachers, as well as foreigners. They have lived in a multicultural context, and through multilingualism have experiences that some may suggest should counter the adoption of raciolinguistic stereotypes. In addition, acquisition of the Chinese language of this group of students has long been a target for research, while their English study experiences have long been ignored. Students from Outer-circle countries and regions (hereinafter referred as SOCs) have been targeted as the participants. To test the extents of SOCs’ linguistic or racial stereotyping against English teachers, three detailed objectives were set for this study:

1. to investigate students’ linguistic and professional assessment of English teachers of the three Circles;
2. to examine students’ background and attitude homophily perception of their teachers and their correlation with the assessment; and
3. to observe the effects of gender homophily on student perceptions on the teachers.

## 2. Materials and Methods

### 2.1. Participants

This study was conducted in a university located in the southern part of Fujian Province, China, which has long been a destination for return for overseas Chinese and thus attracting a great number of Chinese heritage students. We used a broad outreach to 700 Chinese international freshmen using the Chinese social media app Wechat (Version 8.0). These students were in their last English class of their first academic year in university. A convenience sample of 48 females and 48 males agreed to be part of the study.

The sample consisted of students who came from Singapore, Malaysia, India, Hong Kong the Philippines, South Africa, etc., and “returned” to China’s mainland to “seek their roots”. These particular ESL countries and regions consist of citizens representing multiple languages and races within their communities. The participants were verified as trilingual speakers who use English as their second language and have had NS as well as NNS teachers previously, and had before been required to acquire a relatively higher command of Mandarin Chinese (HSK level 5 and above) for tertiary education. They also reported speaking fluent Malay, Filipino, Hindi, Hakka or Cantonese, and having had NS teachers for English courses in primary or secondary education. They were all between the ages of 18 and 20, and only expressed binary gender identity, with no one choosing a third gender preference. They majored in 11 different disciplines, including science, engineering and humanities, and were instructed by Chinese EFL teachers for their English courses.

### 2.2. Instruments

A mixed study method is used, with a 3-section online questionnaire for quantitative data and a face-to-face interview for qualitative results. The questionnaire is prefaced with an introduction and begins with a first section that collects the students’ demographic data in terms of age, gender, and university details. Both sub-questionnaires, i.e., the second and third sections, feature homophily scales and teacher assessment scales, respectively, both having passed the Cronbach test (Cronbach’s α = 0.824 and 0.853). For each of the items in the 2 and 3 sections, a five-point Likert scale (5 = strongly agree; 4 = agree; 3 = neutral; 2 = disagree; 1 = strongly disagree) is used to elicit students’ answers. Teachers are classified into 3 groups for student evaluation: *NS teachers* (in your former experience), *teachers from your own nationality/race* (in your former experience), and *Chinese teachers* (in your freshman year experience). For the gender effect tests, each group of both teachers and students is further divided into sub-groups of male and female. The questionnaire is distributed using questionnaire app Wenjuan-Xing, which can be further connected with the app Wechat for distribution and data collection.

#### 2.2.1. Homophily Scales

The first section of the questionnaire is homophily scales. This study copies and modifies the homophily scales of McCroskey et al. (2006) [33], which has a total of 17 items employed for the attitude dimension, and 10 items for the background dimension. SOCs are invited to rate their perceptions of the level of homophily with their Chinese EFL teachers who are also classified by two genders.

#### 2.2.2. Teacher Assessment Scales

The second section of the questionnaire is teacher assessment scales. Following and modifying Kang et al. (2015) [34], the teacher assessment scales include specific items pertaining to linguistic competence and to instructional competence, namely teachers’ *affinity*, *teaching competence*, *English competence*, *accent standardness*, and an *overall evaluation*.

#### 2.2.3. Qualitative Interviews

To further explore perceived homophily, 8 students (4 males, 4 females) who volunteered to be interviewed participated in semi-structured interviews. They are between the ages of 18 to 20 and are from Hong Kong, India, Malaysia, Singapore, and the Philippines. Six of them major in social sciences and two are engineering majors. To decrease any undue influence by having the faculty interview the students, a peer is recruited to interview each of the students in their residence halls in Chinese. This occurred a week after the questionnaires were completed. All interviews are semi-structured and follow a common interview protocol, carried out personally and individually and recorded with a digital audio recorder. Two questions are asked with follow-up prompts to gain further insights into their initial responses, such as “*can you give us examples”*, or “*do you have anything else to add”* and “*why do you think that…”*:

*1*. *Do you fully comprehend the content of the questionnaires, including background, attitude, gender, race, and regional homophily?*
*2*. *From these five perspectives, do you have any specific thoughts or other supplements on the teachers you had (from these three different regions)?*

The first question addresses the concept of homophily and raciolinguistics, while the second allows deeper conversation regarding the topics in the first question. The interviews are conducted in Chinese, recorded and then later transcribed. They take place in a safe and relaxed location at the participants’ residence hall.

### 2.3. Procedure

Following the approval of our study by the institutional review board of the first and third author’s college, students participated in the survey at the end of their last English class of the freshman year. Before the online questionnaire was distributed from the questionnaire app Wenjuan-Xing to the social media app Wechat, they were given the opportunity to choose whether or not they were willing to continue with the questionnaires. They were also asked if they would like to participate in an interview. Those 8 who were interested participated.

### 2.4. Data Analysis

Data analysis occurred in two steps. The first involved analyzing of the quantitative data from the instruments, while the second involved a content analysis of the 8 interviews. Quantitative data: Questionnaire results were analyzed using one-way ANOVA, correlation analysis, and paired-sample tests in the SPSS Statistics 26.0 software package. First, one-way ANOVA tested to what extent the SOCs rated teachers of the three Circles differently. Second, correlation analysis was adopted to see the correlation between the SOCs’ homophily perception and their teacher assessment results. Finally, the paired-sample tests were implemented to address the gender differences between male and female SOCs on their ratings of the teachers.

Qualitative data: Semi-structured interviews were conducted in the study to triangulate the quantitative results and to obtain a fuller and deeper understanding of the perceptions and feelings of the participants. The recorded interviews were transcribed, read through, and double checked by the first and the third authors. The transcripts were analyzed for patterns and then themes. A constant comparative method, etic approach, was used to note similarities and differences within each of the 3 categories of teachers and of the 2 genders of SOCs across all interviewees. An etic approach was used to examine the data initially in regard to the two questions on gender homophily and racial discrimination. Then, the data were analyzed using an etic approach that applied the factors of *background*, *attitude*, *gender*, *race and regional homophily,* according to the concepts within the scales that included *affinity*, *accent standardedness*, *teaching competence*, and *overall evaluation*. These categories were applied to the students’ responses to note if these particular categories were found, and, if not, what other patterns were noted.

## 3. Results and Findings

### 3.1. Significant Racial/Geographic Differences in the Teacher Assessment

The results of One-way ANOVA tests and repeated contrast measures are reported in Table 1. In the five dimensions of teacher assessment, there exist statistically significant differences across the three groups. For *affinity* (*F* (2, 382) = 13.396, *p* = 0.000), *teaching competence* (*F* (2, 382) = 29.121, *p* = 0.000), *accent standardness* (*F* (2, 382) = 96.449, *p* = 0.000), *English competence* (*F* (2, 382) = 74.343, *p* = 0.000), and *overall evaluation* (*F* (2, 382) = 12.749, *p* = 0.000).

**Figure 1:**
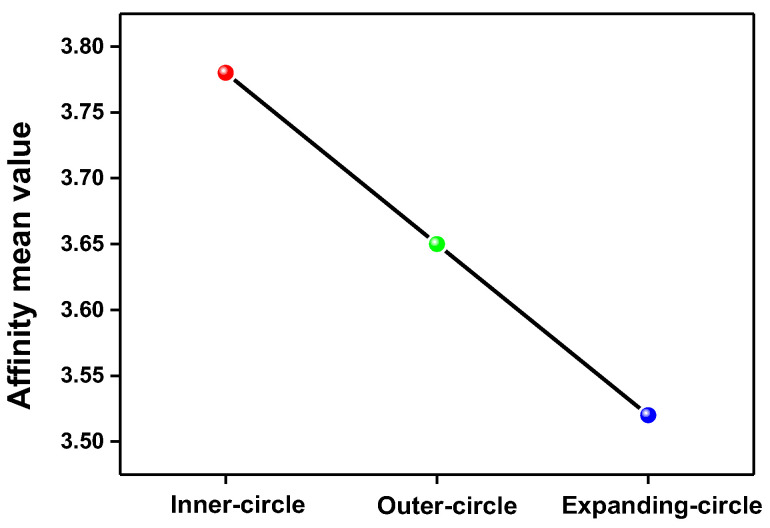
Comparison of affinity assessment across three teacher groups.

These results show that the SOC assessment of teachers of the three Circles is in line with the linear order of the concentric Circles. The students still regard Inner-circle teachers superior to their peers of the Outer-circle and the Outer-circle superior to those of the Expanding-circle. An NS preference or a racial/geographic difference prevails as a hierarchy in SOCs’ perception of teachers. Figure 1 illustrates the significant differences of SOCs’ scoring on *affinity* across the three groups of teachers.

### 3.2. Significant Background Heterophily and Slight Attitude Homophily Effects

The Pearson’s two-tailed tests (as in Table 2) indicate that *attitude homophily* has extremely low and positive correlation with the *overall evaluation* and *teaching competence* (*r_attitude−evaluation_* = 0.109, *p* < 0.05; *r_attitude−teaching competence_* = 0.153, *p* < 0.05). However, *background homophily* has a medium level or a slightly negative correlation with the dimensions of *accent standardness*, *English competence*, and *overall evaluation* (*r_background−accent_* = 0.263, *p* < 0.05; *r_background−English competence_* = 0.364, *p* < 0.05; *r_background−evaluation_* = 0.328, *p* < 0.05).

These results show that the homophily factor has weak albeit some correlation with teacher assessment. This is especially the case with attitude homophily, in which there is no distinctive difference across teachers from the three Circles, as can be seen in Table 1. The negative correlation between background and three of the five teacher assessment dimensions revealed a background heterophily, which is in accordance with the significant distinction among the three groups of teachers.

### 3.3. Significant Gender Heterophily of Male Students

The paired-sample tests (Table 3) revealed a gender heterophily of male students on the dimensions of *teaching competence* (*t* = −2.296, *df* = 143, *p* < 0.05), *accent standardness* (*t* = −2.845, *df* = 143, *p* < 0.05), and *overall evaluation* (*t* = −3.546, *df* = 143, *p* < 0.05); they assign much lower scores to male teachers than female teachers. On the dimensions of *affinity* and *English competence*, the differences are not obvious (*t* = 0.779, *df* = 143, *p* < 0.05; *t* = −1.699, *df* = 143, *p* < 0.05).

Female students do not rate male and female teachers noticeably differently. Despite minute differences regarding *teaching competence* (*t* = −1.665, *df* = 143, *p* = 0.05), *affinity* (*t* = −0.610, *df* = 143, *p* > 0.05), *accent standardness (t* = 1.227, *df* = 143, *p* > 0.05), and *overall evaluation* (*t* = −3.546, *df* = 143, *p* > 0.05), they actually even assign the same scores for *English competence* (*t* = 0, *df* = 143, *p* > 0.05).

The results derived from questionnaires show that male students favor female teachers while female students do not show gender preferences; these findings are also supported by qualitative data collected from the interviews.

In the next section, the results from the qualitative interviews are presented. These interviews were analyzed using the concepts from the instruments, as well as the two research questions.

### 3.4. Qualitative Findings: An Addition That Mitigates the NS Preference

Two themes can be concluded from the qualitative data. The first is the support of the male SOC gender heterophily, as discussed in the previous section. Gender homophily was also noted in the qualitative interviews with male students favoring female teachers, and the female students expressing no gender distinction. For example, two male students stated the following:

“*Chinese male teachers usually have accent problems, but the lady teachers are usually speaking beautiful English.*”

(Male SOC 2)

“*We all know the lady teachers are more careful.*”

(Male SOC 3)

Meanwhile, a female student expressed lack of preference: “*Male teachers may not explain the details of English as well as the lady teachers do, but they are just good anyway.*”

(Female SOC 1)

These findings support the quantitative findings as well regarding gender homophily.

The second key pattern is the support for the NS preference, i.e., racial/geographical heterophily and for the Three Circle theory in many aspects. This brings an addition to the NS preference. In the perceptions of Chinese heritage SOCs, native English is still considered “best”, ESL areas are considered secondary, and English spoken by Chinese people in the EFL region of China is considered the lowest among the three. This recognition of NS preference can be seen in the following quotes:

- “*Hong Kong’s education is more or less related to the UK’s so our (HK) English teacher is also good.*” (Male SOC 4)
- “*Not all, but most teachers in Singapore are excellent English speakers, close to native.*” (Male SOC 1)

These findings support the research regarding the NS preference in the Inner-circle while expanding our understanding that the third circle of Chinese EFL teachers face more prejudice than others. The next section speaks to the particular factors examined in the instruments.

In regard to the first factor, *affinity*, participants stated that stronger criticism is imposed on both Inner-circle and Chinese teachers. This is reflective of the racial and geographical heterophilia discussed earlier. Comments focused on the quality of the relationship between teacher and students as represented by the two comments below.

- “*The western countries are more open… the teachers are like friends to the students. It’s so not the case in Asia, coz we value ‘zun shi zhong dao (respect your teachers and value the Way)’, so the teacher–student relationship is always rigid. It’s in our history.*” (Male SOC 4)
- “*Most teachers are good already. But the mainland has a huge number of students so the teachers only pet those who are good with study… the poor students feel ‘abandoned’.*” (Female SOC 4)

These comments can also reveal the participants’ perspective as foreign-born Chinese students returning to the homeland. But they still reveal a prejudice against their Chinese faculty.

The next factors, *accent standardness* and *English competence*, were the two qualities that also reflect the preference for native speakers, but increase allowance for Chinese teachers who have advanced education in an English-speaking country. The two quotes below illustrate this point. The first quote emanates from outside of China, but does show a bias against the Chinese English faculty.

- “*I won’t say my teacher in Singapore is as good as the Canadian. She received her MA in Canada and she said she was afraid that her English is not as good as the Canadian. But she’s still better than Chinese teacher. Lady teachers usually don’t have any problems.*” (Male SOC 1)
- “*European and American teachers are of course the best, for they are native. Our teachers back in the Philippines are also having standard accent; we use American education system in my hometown; it can’t be as good as the American or British for sure. I can understand the Chinese teachers’ accents. No problem with that.*” (Female SOC 1)

Though not a direct bias in this second quote, she still points to the preference for an “American accent”. In both quotes, home experiences have shaped a preference, but also slight bias against the Chinese English teacher.

The next factor, *teaching competence*, was a theme that addressed a preference for a more “Western-speaking approach” over rote-learning assignments. For example, the first quote is reflective of a preference for the American style of classroom teaching, while the second quote notes the increased emergence of this approach in some of the Chinese-teacher-taught courses, and the third is a rather positive comment on the special expertise of Chinese EFL teachers.

- “*Chinese teachers always ask us to memorize English words or even paragraphs. That’s silly and annoying. I am sometimes under pressure. Back home (in Malaysia), we always study English so we are more natural with the language. And English is like Malay in many ways. We use English every day. I think the native teachers will have some easier ways to teach. Americans are known for their flexible classes, right?*” (Male SOC 2)
- “*Mainland teachers are different than before now. They are not bad. They must have gone through the language study as a foreign language themselves, so they know what’s difficult for us, and they how to teach others.*” (Male SOC 3)
- “*The English teachers will mark at places where you made a grammatical mistake, but they didn’t make me understand why. Now the Chinese teachers are very good at giving a lot of examples to make grammar understandable. It’s their strength.*” (Female SOC 1)

Again, these comments could also reflect the participants’ role as international students who have experienced different instructional strategies back home. All international students must make the same adjustments, but some of the participant above have points of reference due to previous experiences with Chinese teachers.

The last factor concerns the *overall evaluation* of the teachers. Again, this reflects that the students are accepting of the Chinese teachers, but there is still a preference for native-speaking English teachers. The comments below reflect this qualified acceptance of the Chinese teachers.

- “*I think all teachers are just good and most of them are just kind. Our Chinese teachers may not speak as well as the native teachers, but they can make it to teach in the university, so it already tells us that they are good enough.*” (Male SOC 3)
- “*There is no high or low about the teachers. Everyone has his or her strengths and weaknesses. Of course, native will be best. It’s English anyway. Actually, It’s not the teachers that matter, it’s that I don’t love studying that much. Haha.*” (Female SOC 3)
- “*Real difference lies in the diploma the teachers have. In India I went to an international school so most of my teachers had high diplomas. Here in China, since we are talking about university teachers, who should have PhD degrees and won’t be too poor, it’s easy to come to this conclusion—they are qualified for us. My friends told me that because our tuition is higher than that of local students, the school gave us the best teachers.*” (Female SOC 2)

So far, the formation of a three-layered hierarchical opinion is very evident, supporting the native speaker fallacy in many measures. However, a third and also most important theme is the discovery that, despite all the differences, Chinese teachers are seen as “*good enough*”, “*just good*”, “*best teachers*” and “*qualified*”, as can be seen from the above underlined statements. These comments though are coached in the multiple experiences of the participants with NNS and NS English teachers while back home. Despite their multicultural experiences and apparent acceptance of their Chinese English teachers, as discussed earlier, they still carry similar biases to their Chinese native colleagues, as evidenced in previously discussed research.

## 4. Discussion

**Figure 2:**
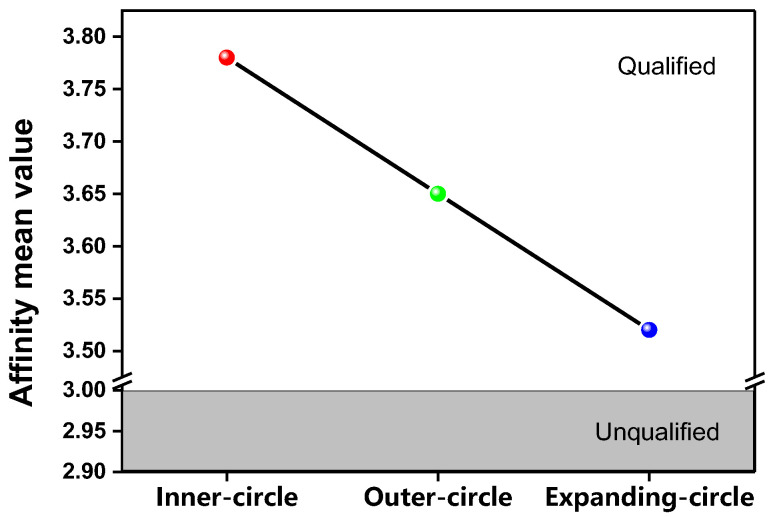
SOCs’ perception of teachers from three groups.

This study features those Chinese heritage undergraduates who feature a trilingual ability and multicultural experiences. With a Three-Circle and mixed study design for these SOCs, students show more homophily and more objective, less biased perceptions in regard to the binary NS/NNS distinction; they are more accepting of the Chinese EFL teachers on certain scales. In this study, the dichotomy is both explicit and more importantly challenged and expanded. It is explicit in that the NS preference is still seen to exert strong power over the students. It is expanded in two senses: First, the dichotomy has been expanded into a progressive order of “*Inner > Outer > Expanding*”, supporting Kachru’s WE theory while stressing the differences among the three groups; Second, the Expanding-circle teachers, especially mentioned as university teachers, are also acknowledged as “*qualified and good enough*”, despite the inferiority in ratings compared to those in the other two circles. As shown in Figure 2 below, equation is now expanded into “*Inner > Outer > Expanding = Qualified > Unqualified*” (Figure 2). Therefore, better than an NNS bias, “stereotyping” would be a more appropriate description. The homophily principle has modest influence on the results. Homophily perception and the effects of multilingual and multicultural experiences might contribute greatly to these findings.

### 4.1. The Effects of Homophily

The teacher assessments of the Outer-circle region participants of our study portray a homophily-mitigated NS preference, and the negative correlation between background homophily and scorings on *accent*, *English* and *teaching competence* measures accord with their high rating for Inner-circle teachers. Further, sharing similar backgrounds, the SOCs rate the ESL teachers from their country of origin lower than the NS teachers but higher than the Chinese teachers. This result is rather similar to that from the study in Thailand in which the Thai undergraduates rated UK, US and Thai English speech significantly higher than other Asian forms of English (Chinese and Indian) for competence, warmth, and perceived positive attitude of the speaker, revealing a clear background homophily [57]. More intriguingly, language learners as young as five years old can exhibit an even closer pattern of homophily preference than ours [9]. When invited to choose their “favorite” teachers, these young respondents choose NS over NNS teachers, and Canadian teachers over NS Australian and English teachers. This homophily extent might also be exercising some influence on the positive recognition and acceptance of the Expanding-circle teachers by virtue of their having the same “blood” as these “roots-seeking” overseas Chinese students. This acknowledgement of a shared Chinese background is also conspicuous in Table 1 on the Background dimension, where the SOCs showed a significantly different order of “*Outer > Expanding > Inner*” from almost all the other assessment factors.

While background homophily might have some effects on teacher preference, attitude homophily has only a slight correlation with ratings. This is further confirmed by the qualitative data that “*most of them are just kind*” (Male SOC 3). Gender heterophily is detected with male students. Binary gender is one of the key variables in teacher–student interactions [58] (p. 6); girls usually perform better in language acquisition, perhaps because they ignore the personal attribute differences among teachers and focus more on the differences or changes within languages [59] (p. 202). Some scholars attributed girls’ better performance in language acquisition to the prospective bias against females in the job market that they are going to confront when they grow up [60] (p. 59). Conversely, feeling less pressure about career prospects, the male students easily transfer their focus from linguistic details to non-linguistic factors, for example, to the gender of teachers. In addition, male students care more about language output [61] (p. 20), meaning, compared to studying grammar or phonetic symbols of another language by themselves, they usually prefer practicing the language by chatting with people, and chatting relies much on interpersonal communication and with whom they are communicating, which in a classroom setting is the teacher.

### 4.2. The Effects of Multicultural Experiences and Multilingual Interventions

When probing into students’ language attitudes in educational contexts, especially in recent years, Lee and Du (2021) [13] detected that the conclusion is usually more complicated than mere NS preference, which is a finding in agreement with this study but contrasting with most investigations of US undergraduates. As suggested by the title, “*She does have an accent, but…*” [4], a mitigated NS preference conclusion is produced. Also, NNS students exhibit changes in their attitudes towards NS and NNS teachers compared to their forerunners [1]. According to some researchers [34,41,62,63], multicultural experiences that come with globalization have been used to justify the changes, and multilingual interventions have been implemented in and out of classrooms and language labs to manipulate the NNS stereotype. It makes this particular group of participants unique in that they come with strong multicultural, multiracial and multilinguistic experiences. The only factor to perhaps further consider could be their role as international students, despite their Chinese heritage.

#### 4.2.1. Sociocultural Perspective: Experiences and Interventions

Multicultural experiences have shaped student perceptions. In this current study, the participants are invited to rate the teachers based not on their imagination, such as in the matched guise studies [12,15], but on their actual experiences. In this case, their lived experiences might account for their judgement of performance, and those who are without multicultural experiences can only judge from imagination or stereotypes so they do not make good participants in an actual classroom setting for teacher assessment [64] (pp. 37–38).

A recent online survey in Australia collected university students’ perceptions of speeches made by either a Chinese guise or a Caucasian guise, and there appear no statistically significant differences between student responses. Because this survey was implemented in Australia, the scholars decided that this difference from Rubin’s findings [6], though both in an NS country, should primarily be attributed to the multilingual and multicultural composition of Australian universities [40]. Also in Australia, students judged that an NNS accent is “attractive” [65]. Studying and travelling abroad also make a difference. Perhaps closest to the current study is an investigation on the sojourning Japanese EFL learners’ perceptions of World Englishes: without veering from the NS norms, when the Japanese youth are informed about the ASEAN nation’s prestige worldwide, they embrace the Outer and Expanding circles better and their notion of Englishes becomes broader [63]. Before actually “opening up” to other cultures, the sojourning Japanese EFL learners are dominated by NS preference and indifferent to the possible “attractiveness” of other varieties of English [55].

Active classroom teacher–student interactions throughout the academic year might also contribute to the positive feedback from students, especially on the scales of *teaching competence* and *overall evaluation*. Researchers have proven that multicultural and linguistic interventions work efficiently to moderate the NS preference for both NS and NNS learners. Kang et al. [34] reported two studies in which the US undergraduates engaged in cooperative problem-solving exercises with NNS teachers. Results show that with exposure to structured intergroup contact, students subsequently rated NNS teachers higher in instructional competence and comprehensibility.

#### 4.2.2. Political Perspective: Successful Chinese Policies

In the case of young SOCs in this study, it is convincing enough that their less negative and more objective and sometimes even positive attitude towards the Chinese EFL teachers—which is manifest especially in their interview comments on the *teaching competence* and *overall evaluation*—is the result of a one-year exposure to multicultural and multilingual environments. In addition, an important factor that should not be underestimated is the Chinese culture identification policies designed and implemented by the Chinese government and universities. In year 2021 alone, there were approximately 100 National Social Science grants disbursed (ranging from JPY 50,000 to JPY 200,000 each) targeting both the improvement of Chinese cultural integration and also the relevant research of the overseas Chinese and Chinese people of Hong Kong, Taiwan and Macao. In university classrooms, there are both English and Chinese medium courses that introduce Chinese wisdom, culture, traditions and history. Students share both content courses and language courses with local students in a hope to “merge” them better, thus providing a considerable amount of cross-cultural interaction. Outside the classroom, universities have organized “root-seeking” cultural journeys all around China for Chinese heritage students. There are also a variety of culture- or language-oriented competitions in which students are encouraged to join free training courses and compete in demonstrating Chinese knowledge acquisition, such as in Chinese calligraphy, painting, and opera competitions. For all these extra-curricular activities, the Chinese government allots special funds, recognizing that China values the experience of these students. In daily lives, different from US universities where students are encouraged to live off campus, Chinese international students are offered on-campus dormitories where they actually share a living experience with the local students. All this friendliness in Chinese policy helps to successfully achieve a more friendly and less biased attitude within SOC students towards China in general, and thus an improved teacher–student relationship more specifically.

### 4.3. The Raciolinguistic Effect of NS Preference and the WE Theory

Despite all the above discussions and the emphasis on the working mechanisms behind the modest mitigation of stereotyping of the SOCs—compared with many other surveys on student–teacher assessment in extant literature (as mentioned in the previous review of the literature)—what cannot be ignored by the researchers and what both the quantitative and qualitative result are revealing is still the hierarchical descending assessment of “*Inner > Outer > Expanding*”. This result, which supports the WE theory and brakes the NS/NNS preference, is still exhibiting raciolinguistic features: if both Outer and Expanding circles are bracketed together and termed “non-native”, the NS preference becomes obvious. This is especially obvious in Figure 1 and in the interview answers regarding *accent standardness* and *English competence* that “*(his teacher in Singapore who) received her MA in Canada…said she was afraid that her English is not as good as the Canadian*” (Male SOC 1) and that “*European and American teachers are of course the best*” (Female SOC 1). The quoted Singapore teacher’s self-evaluation is exactly in line with the psychological inferior complex that Fanon [66] concluded about the French-speaking Black person who often developed a feeling of inferiority and is always reminded that they can never be fully French. An intriguing coincidence is that both the teacher and the Black speakers are from places that have been previously colonized for a long time. According to raciolinguistics, this is actually not a coincidence because white supremacy is still exerting its effects through the vehicle of language usages (among many others) in the postcolonial era [11], and this echoes the proposition that race is not fixed and predetermined but rather reiterated out of continuous and repeated discourses [10].

A closer look at the backgrounds of the eight SOC interviewees displays that they are all from previously colonized places: Hong Kong, Singapore, the Philippines, Malaysia and India. The ESL regions are the regions that “have passed through extended periods of colonization” [50] and this is exactly how Kachru defines the Outer circle. However, different from the raciolinguistic perspective, Kachru, though applying this historical colonial influence, is proposing that people take a much more objective judgement and understanding of the actual use of English. Although criticized for “implying ‘correctness’ of the English used by the Inner-circle [51]”, it seems only fair to acknowledge that the World English theory, as the name suggests, is endeavoring to fight against raciolinguistic stereotypes and to promote a worldwide acceptance of Englishes used in all three Circles of the world, which happens to be one of the main purposes of this study. The results, though, might suggest that much more work needs to be conducted by both society and academia, including the students themselves and the EFL professionals.

### 4.4. Suggestions for Chinese EFL Professionals

As the SOCs in this study actually assess their teachers based not on their imagination but on actual experiences, there arises from these results the fact that both the educators’ and the students’ professional/academic development call for more attention from Chinese EFL teachers.

First, though Chinese teachers were rated lower on most dimensions measured in this study, on top of the fact that they also receive positive feedback especially on *teaching competence* and *overall evaluation*, they themselves have made great strides with increasing the number of higher education degrees from overseas and improving English levels. Perhaps, however, based on student feedback (consider “*rote-taking skill*” and “*(not) open*” up enough to the students, etc.), expanding and diversifying the instructional style would help this particular group of students, as well as all students with NNS perceptions, and thus improve the student–teacher relationships, and also advance the levels of learning. Activities like participation and communication in training sessions, self-assessment and peer assessment might be effective to help [67] (p. 600).

Second, collecting and examining feedback from the students is crucial [24]. While the Chinese government and university administrations have affixed much value to the SOCs’ cultural and daily experiences, the quality and experience of their knowledge acquisition has been long ignored and under-researched, except in respect of their Chinese language acquisition. Feedback about academic development and experiences should start to be valued and examined, as stereotypes have been shown to exert negative influence on academic performance and as whole-person development is the ultimate goal of education. Heeding such feedback will help educators to identify their weaknesses and challenges, and also those of their students.

Third, the native speaker fallacy and racial heterophily discrimination can be dangerous to both students and teachers. Not only does it contribute much to the NNS movement in the TESOL discipline [68], but it can also induce the actual acts of isolation or persecution within a minority individual in a multicultural campus [21]. Taking into consideration the prevailing NS preference in the results, educators and curriculum designers might consider providing some courses introducing the varieties of World English so that when meta-cognition is stimulated, students may be conscious of and may confront underlying yet potent racial discrimination of which they are unaware or reluctant to admit, as identified in matched guise experiments ([33,35], etc.). Successful examples mentioned in Section 4.2 might work as powerful references.

Fourth, as mentioned earlier, well-designed interventions should be included throughout language courses as they are proven to work efficiently to mitigate NS preference. In fact, even a small amount of intervention can serve well. For example, before presenting the two groups of listeners with the same audio speech made by a single French speaker to rate, Reid et al. (2019) [62] told the experimental group a positive anecdote about a French speaker who speaks “excellent” English and shared critical and negative comments with the control group about another French speaker. Subsequently, the young raters, as in our study, assigned significantly higher ratings to the claimed “excellent” English speaker than to the one whose English was formerly criticized. Meanwhile, in their experiment, the older raters did not show similar manipulated rating results. It is therefore obvious that the younger students are liable to adjust their response by positive intervention. In our study, as young learners, the undergraduates can also be oriented with designed instructions towards more positive knowledge of World Englishes. In Hong Kong, a recent study shows that a one-day intervention of cross-cultural learning suffices it to enhance cultural humility and empathy towards teachers in contexts outside of Hong Kong [69].

### 4.5. Limitations and Future Research

As a tentative study of SOC students, our investigation features several limitations that future research should address. First, the scale of the study is limited: through simple sampling, we only collected students from four Outer-circle countries and Hong Kong (*n* = 96), so future studies might employ participants from more diverse origins. Internet and mobile apps might work as a far-reaching tool for data collection. Second, only the Outer circle has been chosen for the convenience of study: future research might be able to compare student perceptions across the Three Circles. Third, as most matched guise studies yield results demonstrating Reverse linguistic stereotype which may be covert to some learners, future studies might follow the common matched guise design and include listening comprehension tests in the survey so as to examine the potential impact of teacher assessment and homophily on language acquisition performance.

## 5. Conclusions

The Chinese heritage undergraduates’ English acquisition processes, especially the ways in which it is affected by their perceptions of English teachers of the Three Circles, is under-researched. As this diaspora of students spread globally, it is hard for scholars to target and design a study. In fact, Chinese heritage undergraduates are actually very typical language learners possessing trilingual abilities and multicultural experiences, and they are under special care of the Chinese government. These features help them achieve an attitude more flexible and tolerant than a simple NS/NNS dichotomy might suggest, thus signaling a reasonable student–teacher relationship. This study is one of the first steps to investigate the students’ assessment of English teachers according to the Kachruvian stratification of Three Circles, and its results mitigate the NS/NNS stereotype and support the WE theory with statistical evidence. With the increasing trend of globalization and cross-cultural exchanges and with the unremitting efforts of educators and researchers, it is promising that more students and teachers will be less subject to the native speaker fallacy and raciolinguistic stereotypes and will adopt a more objective and positive attitude towards qualified English teachers of all races and regions.

## Figures and Tables

**Table 1:** Comparison of teacher assessment and homophily ratings across three teacher groups.

Tested Dimensions	Expanding Circle (EC)		Outer Circle (OC)		Inner Circle (IC)		Sig.	F	Repeated Contrasts
	*M*	*SD*	*M*	*SD*	*M*	*SD*		(2, 382)	
Affinity	3.523	0.899	3.647	0.842	3.78	0.754	0.000	13.396	
Teaching competence	3.358	0.655	3.356	0.713	3.694	0.792	0.000	29.121	
Accent standardness	3.030	1.087	3.080	1.132	4.190	1.028	0.000	95.449	IC > OC > EC
English competence	3.390	1.053	3.440	1.071	4.250	0.976	0.000	74.343	
Overall assessment	3.510	1.028	3.550	0.986	3.830	0.985	0.000	12.749	
Background homophily	2.934	0.615	2.878	0.669	2.717	0.605	0.000	10.399	EC > OC > IC
Attitude homophily	2.941	0.559	2.915	0.567	2.926	0.478	0.880	0.115	No significant difference

**Table 2:** Correlations between homophily and teacher assessment items.

Homophily	Accent Standardness		English Competence		Overall Evaluation		Teaching Competence		Affinity	
	Pearson Correlation	Sig. (2-Tailed)	Pearson Correlation	Sig. (2-Tailed)	Pearson Correlation	Sig. (2-Tailed)	Pearson Correlation	Sig. (2-Tailed)	Pearson Correlation	Sig. (2-Tailed)
Background	−0.263 **	0.000	−0.364 **	0.000	−0.328 **	0.000	−0.001	0.981	−0.054	0.195
Attitude	0.057	0.172	0.059	0.160	0.109 **	0.009	0.153 **	0.000	0.034	0.413

** Correlation is significant at the 0.01 level.

**Table 3:** Comparison of two genders of SOCs’ ratings on two genders of teachers.

Students	Measures	Male Teachers		Female Teachers		*MD*	*t*	Sig.	Repeated Measures
		*M*	*SD*	*M*	*SD*				
Male	Teaching competence	3.377	0.730	3.444	0.738	−0.677	−2.296	0.023 *	Female > Male
	Accent standardness	3.230	1.227	3.430	1.250	−0.201	−2.845	0.005 *	
	Overall evaluation	3.320	1.035	3.540	1.070	−0.222	−3.546	0.001 *	
	English competence	3.510	1.165	3.610	1.153	−0.104	−1.699	0.092	No significant difference
	Affinity	2.995	0.554	2.979	0.596	0.016	0.779	0.437	
Female	Teaching competence	3.510	0.741	3.545	0.741	−0.035	−1.665	0.098	No significant difference
	Accent standardness	3.560	1.163	3.520	1.171	0.042	1.227	0.222	
	Overall evaluation	3.820	0.913	3.830	0.926	−0.007	−0.156	0.877	
	English competence	3.830	1.047	3.830	1.026	0.000	0.000	1.000	
	Affinity	3.748	0.881	3.774	0.880	−0.026	−0.610	0.543	

* *p* < 0.05.

## Data Availability

The data supporting the findings of this manuscript are available from the corresponding author upon reasonable request.
